# Luminescent Ink Based on Upconversion of NaYF_4_:Er,Yb@MA Nanoparticles: Environmental Friendly Synthesis and Structural and Spectroscopic Assessment

**DOI:** 10.3390/molecules26041041

**Published:** 2021-02-17

**Authors:** T. M. Dung Cao, T. T. Giang Le, Sylvia Turrell, Maurizio Ferrari, Quang Vinh Lam, T. T. Van Tran

**Affiliations:** 1 Faculty of Materials Science and Technology, University of Science, Ho Chi Minh City 700000, Vietnam; ctmdung@hcmus.edu.vn; 2 Vietnam National University, Ho Chi Minh City 700000, Vietnam; lttgiang@hcmus.edu.vn (T.T.G.L.); lqvinh@vnuhcm.edu.vn (Q.V.L.); 3 Faculty of Physics and Engineering Physics, University of Science, Ho Chi Minh City 700000, Vietnam; 4 CNRS, UMR 8516-LASIRe–Laboratoire Avancé de Spectroscopie pour les Intéractions la Réactivité et l’Environnement, Université de Lille, F-59000 Lille, France; sylvia.turrelljones@gmail.com; 5 IFN-CNR CSMFO Lab. and FBK Photonics Unit, Via Alla Cascata 56/C, 38123 Povo, Italy; maurizio.ferrari@unitn.it

**Keywords:** NaYF_4_:Er,Yb, upconversion emission, surface modification, hydrophilic, screen printing

## Abstract

NaYF_4_:Er,Yb upconversion luminescent nanoparticles (UCNPs) were prepared by hydrothermal methods at 180 °C for 24 h. The X-ray diffraction (XRD) and TEM (transmission electron microscopy) images show that the resulting 60 nm UCNPs possess a hexagonal structure. In this work, maleic anhydride (MA) was grafted on the surface of UCNPs to induce hydrophilic properties. The photoluminescence spectra (PL) show upconversion emissions centered around 545 nm and 660 nm under excitation at 980 nm. The luminescent inks, including UCNPs@MA, polyvinyl alcohol (PVA), deionized water (DI), and ethylene glycol (EG), exhibit suitable properties for screen printing, such as high stability, emission intensity, and tunable dynamic viscosity. The printed patterns with a height of 5 mm and a width of 1.5 mm were clearly observed under the irradiation of a 980 nm laser. Our strategy provides a new route for the controlled synthesis of hydrophilic UCNPs, and shows that the UCNPs@MAs have great potential in applications of anti-counterfeiting packing.

## 1. Introduction

Over the past few years, luminescent inks have been used to detect counterfeiting. Outstanding applications of these inks include banknotes, quick response codes (QR codes), barcodes, security documents, drug packaging, and food security [1,2,3,4,5]. The security ink is invisible under visible light; but, upon ultraviolet radiations, printed information can be read. These security inks are mostly based on the down-conversion effect, and primary materials, such as quantum dots (QDs), are used [6,7,8]. In fact, due to their wide band gaps, they easily absorb ultraviolet light. 

CdS and CdSe are commonly used for syntheses, but they are toxic. Moreover, UV radiation is harmful to our health under specific conditions. In addition, the substrates are also sensitive to UV excitations, which leads to a reduction of the contrast between the substrate and the printed sample. Last but not least, UV to visible down-conversion materials and UV excitation sources have become more accessible, making them much easier to duplicate. 

For these reasons, luminescent inks using near-infrared (NIR) excitation are now studied as alternatives. The resulting materials are non-toxic, and the ink can easily glow even on highly luminescent substrates, such as paper and textiles. Near infrared to visible luminescent materials based on the so-called upconversion luminescence are more difficult to duplicate compared to UV to visible materials. Nowadays, rare earth ion (RE) doped luminescent materials are being studied to replace QDs. This direction is due to the abundance of excitation and luminescence wavelengths depending on the type of REs selected [9,10,11,12]. For materials using infrared excitation, Yb^3+^ ions are chosen as sensitizers to absorb 980 nm radiation, while the Er^3+^ ions are activators for visible emissions [13,14,15]. 

In another approach, the luminescent host material is constituted by NaYF_4_ nanoparticles, synthesized by hydrothermal methods with oleic acid (OA) and used as surfactants [16,17,18,19]. The UCNPs produced using this approach are inherently capped with hydrophobic ligands because OA is a fatty acid, which contains an alkenyl group (–HC = CH–). However, applications require nanocrystals to be dispersed and stabilized in the aqueous phase. Thus, the transfer of these hydrophobic nanocrystals into aqueous media becomes a critical issue.

To synthesize high-quality, water-dispersible nanocrystals, there are many polymers being studied, such as poly(methyl methacrylate) PMMA [20,21], poly(acrylic acid) PAA [22,23], and polyethylene glycol PEG [24]. In our study, maleic anhydride (MA) was chosen for hydrophilic properties [25,26], because it can react with oleic acid capped on the surface of nanoparticles. The resulting UCNPs will exhibit dispersibility in water. 

In the maleinization process, MA can be grafted onto hydrophobic ligands bearing alkenyl functional groups to yield succinic anhydride functional groups. There are a few advantages of this new strategy, such as retaining nanocrystalline characteristics, using a simple hydrolysis treatment and reaction conditions for the maleinization of oleic acid, and providing carboxylic acid functional groups that provide good anchors. The UCNPs with hydrophilic bonding on the surface can be successfully applied in many fields. One of them is ink printing, due to the stabilities of UCNPs in water and solvents [27]. Compared to various methods, such as radio frequency identification (RFID) tags [28], inkjet printing [29], and laser holograms [30,31], all of which require high-cost equipment, clean room facilities, and a complex preparation procedure, the screen printing process reveals some outstanding advantages. This method produces images of higher quality than inkjet prints, can be printed on a variety of materials, including glass, wood, textiles, signs, and banners, and can easily be printed on a specific or large area [32,33]. 

There are many formulas for inks, running from complicated to simple in nature [4,9,27,34]. Ink formulations consist of a solution containing the UCNPs, a solvent system, and a polymer that can be dissolved in the solvent system. The solvent system must produce a solution with optimal physical properties (viscosity, evaporation rate, surface tension) suitable for printing. The poly (vinyl alcohol) (PVA) was chosen as the polymer matrix for the preparation of screen printing. Among the various solvents evaluated, ethylene glycol produced stable oxide inks and satisfied the printing conditions. With its simple formula, it was deemed suitable for applications.

## 2. Results and Discussion

### 2.1. Vibrational Characterization of UCNPs

**Figure 1:**
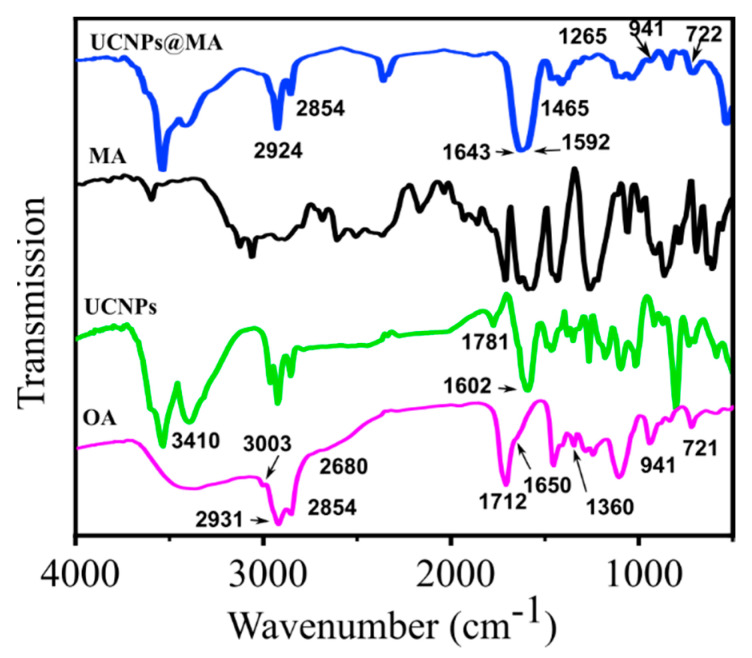
FT-IR spectra of OA, UCNPs, MA, and UCNPs@MA.

The existence of organic bonds on the surface of UCNPs can be demonstrated by the observation of the FT-IR spectra shown in Figure 1.

Fourier transform infrared spectra of OA, UCNPs, MA, and UCNPs@MA nanoparticles are shown in Figure 1. The FT-IR spectra of OA show the ν_as_ (–CH_2_–) and ν_s_ (–CH_2_–) stretching vibrations of long alkyl chains at 2931 cm^−1^ and 2854 cm^−1^, respectively. The O-H vibrational stretching modes of carboxylic acid groups (–COOH) are observed around 3500–2500 cm^−1^, the C = O stretching of a carboxyl group at 1712 cm^−1^, the vibrational modes of carboxylate group (COO^−^) around 1650–1360 cm^−1^, the out-of-plane O–H deformation mode at 941 cm^−1^, and the rocking mode of CH_2_ at 721 cm^−1^. In the spectrum of OA, there is also the appearance of the peak at 3003 cm^−1^, attributed to the olefinic (C–H) vibrational mode in (C = C–H) [35,36]. 

For the prepared NaYF_4_ sample (UCNPs), the appearance of bands at 2931–2680 cm^−1^ confirms the presence of oleate ligand on the nanoparticle surfaces. Moreover, the broad bands located at 3410 cm^−1^ and 1602 cm^−1^ correspond to the O–H stretching and bending vibrations, respectively, of residual molecular water in the samples. It is worthy to note a redshift in the frequency of the C = O and (COO^−^) stretching modes in doped samples, which is the result of the electrostatic attraction and chemical adsorption between the Ln^3+^ ions of nanoparticles and the COO^−^ group of OA in UCNPs [19]. 

The UCNPs@MA spectrum presents the characteristic bands of oleic-maleic anhydride copolymer at 722 cm^−1^, 941 cm^−1^, 1265 cm^−1^, and 1465 cm^−1^, which may be attributed to (CH_2_)_n_, O–H out of plane bending vibrations, C–O anhydride, and CH_2_ vibrational modes, respectively [37]. The broad band around 1643–1592 cm^−1^ is related to the O–H bending and the C = C vibrations of MA. This result confirms the presence of MA bonds on the UCNPs surface. Moreover, the spectra show absorption bands at 2854 cm^−1^ and 2924 cm^−1^, which may be related to C–H symmetric and asymmetric stretching modes, respectively.

### 2.2. Structural Characterization of UCNPs

**Figure 2:**
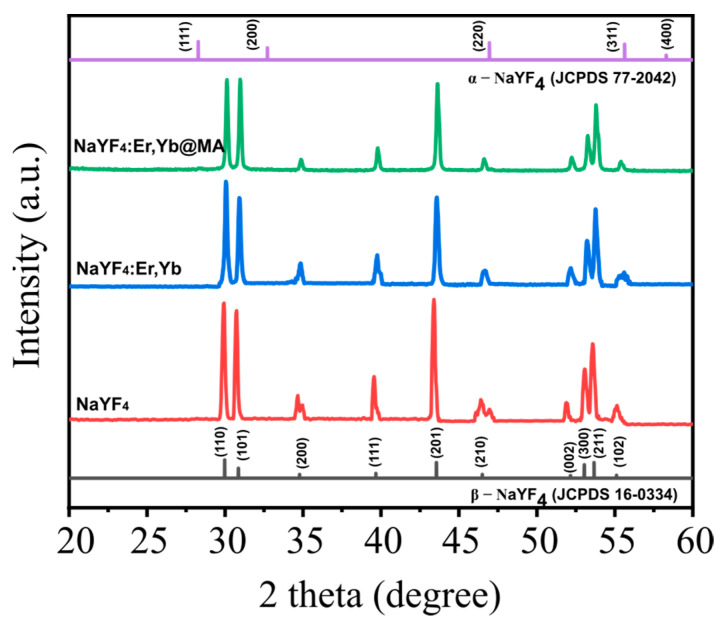
XRD patterns of NaYF_4_ un-doped, co-doped NaYF_4_:Er,Yb, and polymer ligand NaYF_4_:Er,Yb@MA.

Figure 2 shows XRD patterns of NaYF_4_ un-doped, co-doped NaYF_4_:Er,Yb (UCNPs), and polymer ligand NaYF_4_:Er,Yb@MA (UCNPs@MA). The systems have a hexagonal structure, with diffraction peaks at positions 2θ = 17.26°, 29.95°, 30.86°, 34.73°, 36.69°, 43.47°, 46.39°, 52.16°, 53.09°, 53.61°, and 55.24°, corresponding to the lattice faces (100), (100), (101), (200), (111), (201), (210), (002), (300), (211), and (102) of the hexagonal structure of β-NaYF_4_, according to the JCPDS 16-0334 standard. 

XRD patterns of NaYF_4_ crystals show no peaks of an α phase cubic structure, as well as no presence of intermediate or doped phases. Thus, the role of REs has had an insignificant effect on the hexagonal structure of β-NaYF_4_. However, the diffraction peaks of the doped samples shift to larger angles. This implies that the dopants lead to a decrease in the lattice parameters. The substitution of doping ions in the Y^3+^ sites causes a compressive strain, because the ionic radii of Er^3+^ (89 pm) and Yb^3+^ (87 pm) are smaller than the radius of Y^3+^ ions (90 pm). 

The XRD pattern of UCNPs with MA shows the same hexagonal structure of NaYF_4_, and there is no peak characteristic of MA. This result shows that the MA bond is formed on the surface of NaYF_4_ and that it does not exist independently in the sample. 

The calculated crystal size of UCNPs@MA is 60 ± 1 nm, and the crystal lattice parameters are 0.59 nm and 0.35 nm, corresponding to a and c, respectively.

### 2.3. Optical Characterization 

**Figure 3:**
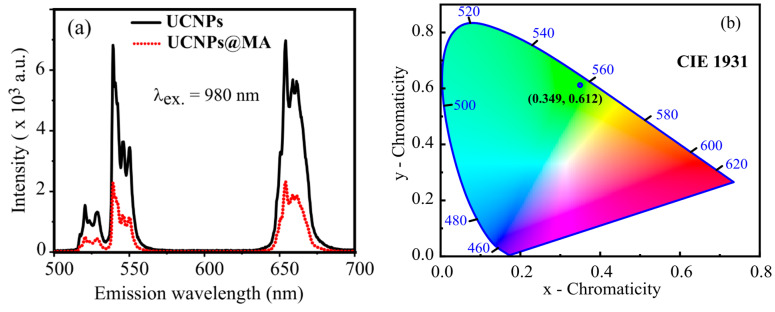
(**a**) UC photoluminescence spectra of UCNPs and UCNPs@MA obtained upon 980 nm excitation; (**b**) the CIE 1931 chromaticity coordinates of the UCNPs@MA.

Figure 3a shows the photoluminescence spectra of UCNP and UCNPs@MA nanoparticles obtained under an excitation of 980 nm. The PL spectra show visible emission of NaYF_4_ co-doped with Er^3+^ and Yb^3+^ ions, with green emission (512 and 560 nm) and red emission (640 and 680 nm) corresponding to the characteristic transition of Er^3+^ ion activators from ^4^H_11/2_, ^4^S_3/2_, and ^4^F_9/2_ levels to the ^4^I_15/2_ ground state, respectively. The effect of hydrophilic polymer bonds on the luminescent intensity of UCNPs is evidenced in Figure 3a. The existence of polymer binding on the surface of the UCNPs results in a reduction of the emission intensity by a factor of about three. The CIE chromaticity coordinates (x = 0.349, y = 0.612) taken from PL data of the UC emission spectra using Origin software show the color of materials to be green, as shown in Figure 3b. 

**Figure 4:**
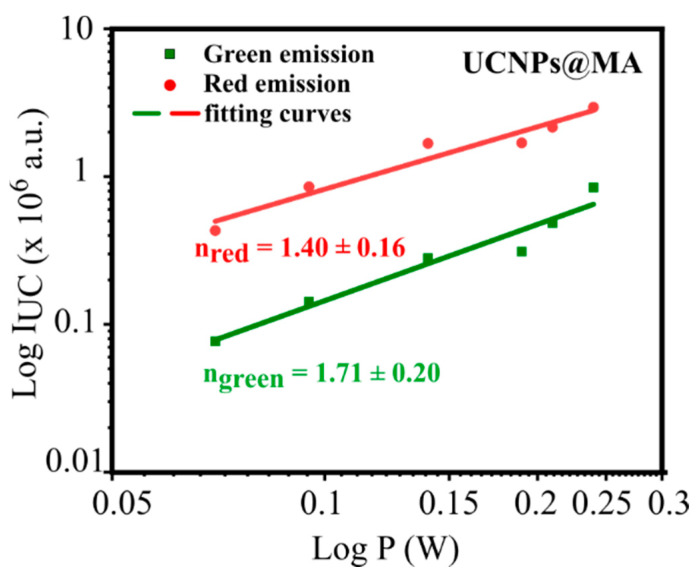
UCNPs@MA upconversion photoluminescence intensity at 545 nm (green) and 660 nm (red) as a function of the excitation power. The excitation wavelength was 980 nm. The solid lines are the curves of a power-law function fitted to the data, which gives the indicated slopes.

To reconfirm the UC emission occurring in UCNPs@MA, the integrated PL intensities (I_UC_) of UCNPs@MA were investigated as a function of excitation power P using the relation I_UC_ ~ P^n^, where n is the number of photons [38]. Figure 4 shows the UCNPs@MA upconversion photoluminescence intensity at 545 nm (green) and 660 nm (red) as a function of the excitation power. The fitting curves indicate the value of n as 1.71 ± 0.20 and 1.40 ± 0.16 for green and red luminescence, respectively.

**Figure 5:**
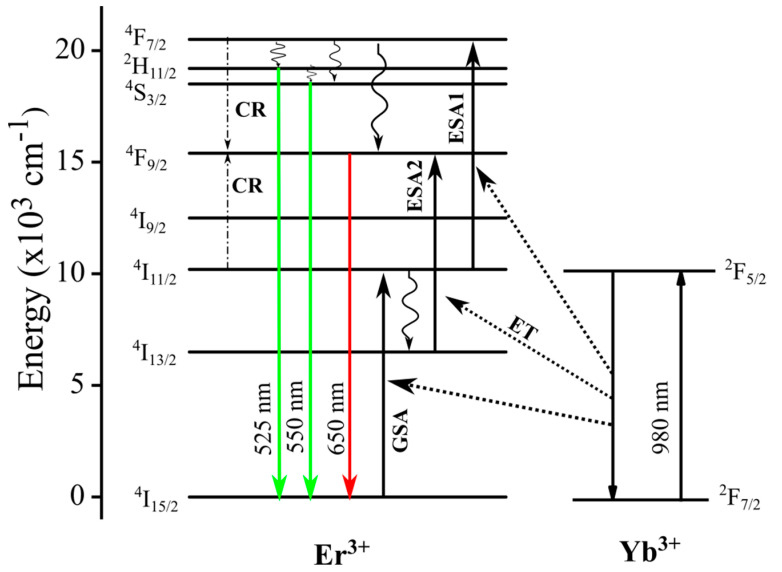
Schematic energy level diagram of co-doped Er^3+^ and Yb^3+^ ions and possible transition processes.

The green luminescence is assigned to a two-photon process. The main two-photon upconversion mechanism is described in Figure 5, where the ground state absorption (GSA), subsequent excited-state absorption process (ESA), energy transfer (ET), and cross-relaxation (CR) in the population of the excited states are indicated. The ^4^S_3/2_ level of Figure 5 is populated by two near-infrared photons. We observe a slope less than two due to competitive processes, such as co-operative energy transfer and cross-relaxation, which are well-described in the literature [39,40]. The slope of 1.4, i.e., close to 1.5, can be explained by a process involving three absorbed photons, which give two emitted photons. The process has been discussed by R.R. Gonçalves et al. [41]. In this process, two infrared photons excite the Er^3+^ ions to the ^4^F_7/2_ state, and one infrared photon promotes another Er^3+^ ion into the ^4^I_11/2_ excited state. After a cross-relaxation process between them, the two ions relax into the ^4^F_9/2_ state, and a red emission occurs [41].

### 2.4. Screen Printing of UCNPs@MA for Anti-Counterfeiting Applications

Due to the outstanding properties of UCNPs@MA, which include hydrophilic surfaces as well as spherical nanoparticles with an average size of 60 nm, which have strong and tunable upconversion fluorescence, this system was used to fabricate environmentally-friendly inks, which could be screen-printed on paper substrates for anti-counterfeiting. 

The UCNPs@MA was dispersed in a solvent mixture of PVA, water, and EG to obtain luminescent ink with a proper dynamic viscosity. By fixing the weight ratio of UCNPs@MA to 0.2 g/mL, a series of UCNPs@MA inks with different solvent compositions were prepared. 

The ink viscosities and the surface tensions with different weights of PVA were measured, and are presented in Table 1. These results show a significant increase in viscosity with the weight of PVA, while the surface tension maintains a value around 60 mN/m for these PVA weights. 

**Figure 6:**
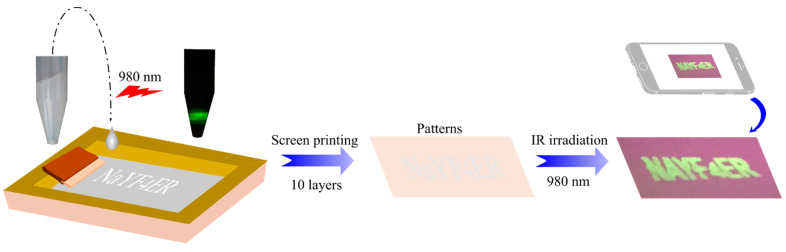
The procedure of screen printing.

An optimized weight ratio of PVA, water, and EG was kept at PVA:EG = 5:1, with a PVA weight of 2 g. These values provided a luminescent ink with the viscosity and surface tension of 68.5 cP and 58.6 mN/m, respectively. This solution was pushed easily through the screen, and displayed clearer images on paper than other PVA weights. The ink solution was scanned through the mesh screen onto the paper, forming 10 layers. The ink patterns were dried under normal ambient conditions for 2–3 min. After screen printing, the designed patterns were invisible under daylight, but clearly observable under the irradiation of a 980 nm LED lamp. This pattern is shown in Figure 6. 

## 3. Materials and Methods

### 3.1. Synthesis of NaYF_4_:Er,Yb Upconversion Nanoparticles with Maleic Anhydride on the Surface 

**Figure 7:**
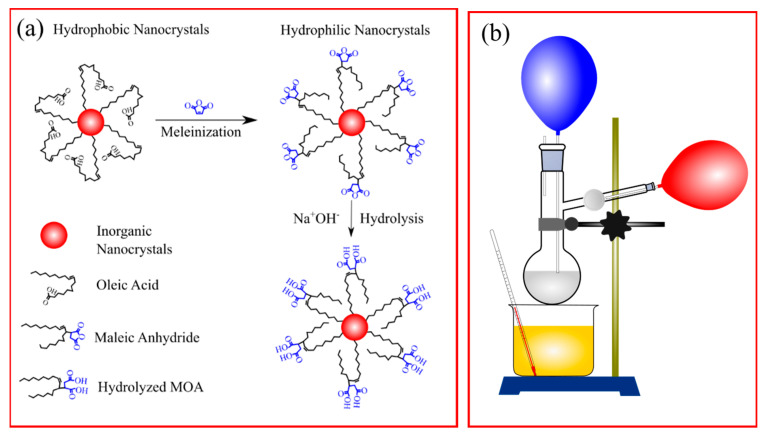
(**a**) Scheme of surface modification of UCNPs by maleic anhydride (MA); (**b**) the experimental apparatus is also sketched.

UCNPs materials were synthesized by solvothermal methods at 180 °C for 24 h. This process is the same as that presented in our previous report [42]. The ratio of rare earth stearate and oleic acid (OA) was RES:OA = 1:24, and doping concentrations of 1% Er^3+^ and 20% Yb^3+^ were chosen for this study. Maleic anhydride (MA, China) was chosen as a polymer to form hydrophilic bonds on the UCNPs surface. Figure 7a describes the surface modification schematic of UCNPs. 

The precursors used for this process were maleic anhydride (MA), benzoyl peroxide (BPO), and toluene (Tol). A weight ratio of UCNPs:MA:BPO = 1:2:5 was employed, and an appropriate volume of Tol was moved into a fixed two-neck flask. Two balloons containing N_2_ gas were used for giving gas out from the surface and inside the solution. An oil container worked as a thermal transfer, as shown in Figure 7b. The modification time was 4 h at 90 °C, with a stirring speed of 300 rpm. After being cooled to room temperature, the NaYF_4_:Er,Yb with MA bonding on the surface (UCNPs@MA) was collected by centrifugation, washed three times with ethanol, and dried at 100 °C for 12 h.

### 3.2. Ink Manufacturing Process

The precursors used for ink preparation included polyvinyl alcohol (PVA), deionized water (DI), and ethylene glycol (EG). The luminescent inks were synthesized in two steps. The first step was to make a mixture of PVA glue using different weights of PVA (1, 2, and 3 g) and 20 mL of DI, stirring vigorously at room temperature for 15 min. The mixture was then heated to 50 °C for 15 min under ultrasonic treatment until a clear solution was obtained. 

The second step was to prepare an ink solution with a ratio of PVA glue and EG as PVA:EG = 5:1. For this procedure, the mixture was vigorously stirred for 15 min at room temperature. At this point, UCNPs@MA was added to the solution and treated ultrasonically for 30 min at 50 °C, in order to ensure that the nanoparticles were well-dispersed in the ink solution. Fluorescent ink solutions exhibited optimal surface tension and viscosity.

The screen-printing frame was used in this work with desirable patterns containing characters with 2 mm width and 5 mm height. An A4 photocopy sheet was used as a substrate for the UCNPs@MA ink deposition during screen printing. No visible images appeared on the paper under normal light, but, under the exposure to a 980 nm excitation, there was the appearance of clear green characters. 

### 3.3. Characterization

Studies of the structural phases were carried out through X-ray diffraction (XRD) patterns under CuKα_1_ radiation (λ = 1.5406 Å), voltage 40 kV, current 40 mA, and tunable 2θ = 20–60° of a D2- PHASER diffractometer (Bruker, Germany). The average crystal diameter D was extracted from XRD data using the Halder–Wagner–Langford method [43]:

(1) $${\left(\frac{\beta^{*}}{d^{*}}\right)}^{2}=D^{-1}\frac{\beta^{*}}{{\left(d^{*}\right)}^{2}}+{\left(\frac{\varepsilon }{2}\right)}^{2}$$

(2) $$\beta^{*}=\frac{\beta .\cos\theta }{\lambda }$$

(3) $$d^{*}=\frac{2.\sin\theta }{\lambda }$$

(4) $$\beta =\;\sqrt{\beta_{\exp}^{2}-\beta_{\mathrm{inst}}^{2}}$$

where β_exp_ is the experimental value of the full width at half maximum (FWHM) of the most intense peak of the sample, and β_inst_ is the instrumental FWHM of a diffraction pattern. Line-broadening β was obtained from Gaussian profile fitting, and a linear fit with terms (β^*^/d^*^)^2^ and β^*^/(d^*^)^2^ was performed, where the slope for the straight line provided the average size D. NaYF_4_ nanocrystals have a hexagonal structure, with lattice constants a and c calculated as:

(5) $$\frac{1}{d^{2}}=\frac{4}{3}\left(\frac{h^{2}+\mathrm{hk}+k^{2}}{a^{2}}\right)+\frac{l^{2}}{c^{2}}$$

The morphology of UCNPs was observed under transmission electron microscopy (TEM) of JEM-400 (JEOL, Tokyo, Japan), with a voltage of 100 kV. For these measurements, the samples were grounded and dispersed in ethanol. A droplet of the resulting fine-powder suspension was placed on a copper microscope grid. Fourier transform infrared spectra (FT-IR) were obtained in the range of 4000 to 500 cm^−1^ with a Vertex70 spectrometer (Bruker, Germany). Photoluminescence (PL) spectra were collected under 980 nm diode laser excitation using an iHR 320 instrument (Horiba, Kyoto, Japan). All measurements were performed under the same conditions at room temperature. 

## 4. Conclusions

Using hydrothermal methods at 180 °C for 24 h, NaYF_4_:Er,Yb nanoparticles with an average size of 60 nm were obtained. The surface modification of NaYF_4_:Er,Yb by maleic anhydride (MA) makes it become a hydrophilic nanoparticle. The visible emissions of these materials can be observed directly with the eyes. A novel and easy ink operating in an upconversion condition has been developed and characterized by its optical, spectroscopic, and structural properties. The core shell structure enables the system to be stable in water and a solvent, thus allowing the use of printing applications.

Finally, the developed ink based on UCNPs@MA emits upconverted green luminescence. This property makes it detectable by human eyes, making this material very competitive for anti-counterfeiting packing.

## Figures and Tables

**Table 1:** Viscosity and surface tension of ink solutions with different weights of PVA.

Weight of PVA (g)	Viscosity (cP)	Surface Tension (mN/m)
1	7.8	59
2	68.5	58.6
3	392.5	62

## Data Availability

The data presented in this study are available on request from the corresponding author. The data are not publicly available due to the rules of research group.

## Sample Availability

Samples of the compounds NaYF4:Er,Yb and their inks are available from the authors.
